# Succinate Modulates Intestinal Barrier Function and Inflammation Response in Pigs

**DOI:** 10.3390/biom9090486

**Published:** 2019-09-13

**Authors:** Xuan Li, Mingyu Mao, Yanan Zhang, Kaifan Yu, Weiyun Zhu

**Affiliations:** 1 Laboratory of Gastrointestinal Microbiology, Jiangsu Key Laboratory of Gastrointestinal Nutrition and Animal Health, College of Animal Science and Technology, Nanjing Agricultural University, Nanjing 210095, China; 2 National Center for International Research on Animal Gut Nutrition, Nanjing Agricultural University, Nanjing 210095, China; 3 National Experimental Teaching Demonstration Center of Animal Science, Nanjing Agricultural University, Nanjing 210095, China

**Keywords:** succinate, intestinal epithelial barrier, intestinal inflammation, pigs

## Abstract

Succinate is a metabolic intermediate of the tricarboxylic acid (TCA) cycle in all aerobic organisms, and is also a vital microbial metabolite in the gut. Although succinate is known to regulate intestinal metabolism and immune function, its role in the protection of the intestinal epithelial barrier function and inflammation is poorly characterized. In this study, we evaluated the effects of succinate on intestinal epithelial barrier function and inflammation in pigs. Twenty-four growing pigs were distributed into three groups (*n* = 8) and received either a basal diet (control group) or the same diet supplemented with 0.1% succinate or 1% succinate. The diet supplemented with 1% succinate led to alterations in the intestinal morphology. We confirmed in vitro that 5 mM succinate treatment modulated intestinal epithelial permeability by increased transepithelial electrical resistance (TEER) in intestinal porcine epithelial cell (IPEC)-J2 cells. Furthermore, succinate treatment increased the abundance of tight junction proteins claudin-1, zona occluden (ZO)-1, and ZO-2 in the jejunum in vivo and in vitro. In addition, dietary succinate supplementation promoted the expression of inflammatory cytokines interleukin (IL)-25, IL-10, IL-8, and IL-18 in the jejunum. Taken together, these data identify a novel role of succinate in the modulation of intestinal epithelial barrier function, which may be a nutritional target to improve gut health in animals.

## 1. Introduction

The intestinal tract, lined by a single layer of columnar epithelial cells, is considered the largest immune organ and plays a key role in gut health. Besides its essential role in nutrient absorption, intestinal epithelial cells, which mainly include absorptive epithelial, goblet, Paneth, and tuft cells, provide a dynamic barrier to inhibit the invasion of the mucosal tissue by luminal commensal bacteria, pathogens, and dietary antigens. The intestinal epithelium, therefore, exerts a barrier function as an intermediary between the body’s interior and exterior. The intestinal epithelial barrier integrity is maintained by tight junction proteins, such as claudins, zona occludens, and occludin [1]. Impairment of the tight junction and increased intestinal permeability are the major features of epithelial barrier disruption, which promote the translocation of luminal antigens into subepithelial tissues, causing mucosal and systemic inflammatory responses [2]. A large number of studies have demonstrated that dysfunction of the intestinal epithelial barrier causes immune system disorders and excessive inflammatory responses in the gastrointestinal tract [3,4]. In parallel, several inflammatory cytokines produced by intestinal immune cells, such as interleukin (IL)-25 and IL-10, can help to attenuate excessive intestinal inflammation and maintain the integrity of epithelial cells [5,6]. Therefore, maintaining the integrity of the intestinal epithelial barrier is essential for gut homeostasis and host health.

Microbial metabolites are capable of modulating the intestinal epithelial barrier and immune response. For example, short-chain fatty acids (SCFAs), such as acetate, propionate, and butyrate, are essential for enhancing intestinal barrier function to maintain mucosal immunity. Treatment with SCFAs has been reported to upregulate the expression of mucin-related genes in intestinal epithelial goblet cells [7,8]. Gastric infusion of SCFAs was shown to improve gut morphology and maintain the intestinal barrier function in piglets [9]. Dietary supplementation with sodium butyrate in piglets promoted intestinal epithelial integrity by increasing the expression of claudin-3, occludin, and zona occluden (ZO)-1 [10]. Consistent with the in vivo study, sodium butyrate also upregulated the relative mRNA expression of tight junction proteins in intestinal porcine epithelial cell (IPEC)-J2 cells [11]. Our previous study found that sodium propionate selectively upregulated the expression of tight junction proteins in intestinal epithelial cells and influenced cellular inflammatory responses [12,13]. A vital role for specific microbial metabolites in the intestinal epithelial barrier and immune response has attracted growing attention.

Succinate is a major microbial metabolite classically described as a key intermediate in microbial propionate synthesis in the gut [14]. It is also an intermediate of the citric acid cycle in hosts. In the intestinal lumen, succinate accumulates to a lesser extent than SCFAs, the concentration of which normally ranges from 1–3 mM (or mmol/kg), but varies depending on the species and sample type [15]. Several studies have demonstrated a crucial role for succinate in the regulation of intestinal inflammation and immunity [16,17,18,19]. Succinate accumulates in inflamed areas and regulates intestinal inflammation and fibrosis by mediating the expression of pro-inflammatory cytokines in macrophages and fibroblasts [16]. Succinate triggers a type 2 immune response in the small intestine by activating chemosensory intestinal epithelial cells called tuft cells, resulting in tuft and goblet cell hyperplasia [17,18]. Schneider et al. showed that succinate was sensed by G protein-coupled receptor 91 (GPR91) in tuft cells, which activates the small intestinal tuft cell-innate lymphoid 2 circuit [19]. Given that the type 2 immune response invokes a protective intestinal epithelial response and the development of inflammation directly affects intestinal epithelial barrier integrity, these findings raise the question of whether luminal succinate sensed by host intestinal epithelial has a homeostatic function to maintain the intestinal epithelial barrier; a question that has not yet been answered.

Thus, we hypothesized that succinate would influence intestinal barrier function and inflammatory responses. We utilized a pig model to evaluate the effects of diets supplemented with succinate on intestinal morphology. The effects of succinate on intestinal epithelial integrity were evaluated in IPEC-J2 cells. We further assessed the expression of tight junction proteins and inflammatory cytokines in succinate-treated groups compared with a control group.

## 2. Materials and Methods

### 2.1. Animals

Pigs were raised and maintained on a local commercial farm. All animal care and experimental procedures were approved in advance by the Animal Care and Use Committee of Nanjing Agricultural University (Nanjing, Jiangsu province, China) in compliance with Chinese guidelines for animal welfare (SYXK(Su)2017-0007).

### 2.2. Experimental Design of the Animal Study

Twenty-four Duroc × Landrace × Large White growing barrows were randomly assigned to three treatment groups (*n* = 8). The treatment groups were (1) the control group, (2) the 0.1% sodium succinate treatment group, and (3) the 1% sodium succinate treatment group. The control group (*n* = 8) was fed a basal diet that consisted of a typical commercial diet. The 0.1% and 1% sodium succinate treatment groups (*n* = 8) were fed the basal diet supplemented with 0.1% and 1% sodium succinate, respectively (Aladdin, Shanghai, China). Each barrow was placed in an individual pen with ad libitum access to feed and water. The experiment period lasted for 28 days.

### 2.3. Cell Culture

The intestinal porcine epithelial cell (IPEC-J2) line was used in this study. The cells were propagated and maintained in Dulbecco’s modified Eagle medium (DMEM/F12; Hyclone, Logan, UT, USA) supplemented with 10% fetal bovine serum (FBS) and a 1% penicillin–streptomycin mixture (GIBCO, Grand Island, NY, USA). The cells were grown in a humidified chamber at 37 ˚C under 5% CO_2_. The cells were propagated or collected every two days when they reached 80% confluence.

### 2.4. Treatment of Intestinal IPEC-J2 cells

Sodium succinate (Sigma-Aldrich, St. Louis, MO, USA) was dissolved in basal Dulbecco’s modified Eagle medium to make the succinate treatment solution. The IPEC-J2 cells were divided into four groups: the control group and the 0.1 mM, 1 mM, and 5 mM sodium succinate treatment groups. The cells and supernatant were collected after 24 h to analyze the mRNA expression of tight junction proteins and the expression of inflammatory cytokines.

### 2.5. Sample Collection

At day 28, all pigs were euthanized, the jejunal and ileum tissues (approximately 2 cm in length) were collected and stored in 4% paraformaldehyde solution for morphological analysis. In addition, a section of the jejunal tissue was immediately washed with cold phosphate-buffered saline (PBS) and stored at −80 °C for quantitative real-time PCR (qPCR) and protein analyses.

### 2.6. Cell Viability

Cell viability was determined by the Cell Counting Kit-8 (CCK-8) assay (Nanjing Jiancheng Institute of Bioengineering, Nanjing, China). Cells (3 × 10^4^ cells/well) were seeded into 96-well tissue culture plates. After succinate treatment, CCK-8 was used to stain the cells for about 2 h. The optical density was measured at 450 nm using a microplate spectrophotometer.

### 2.7. Electrical Resistance Measurements

Transepithelial electrical resistance (TEER) measurements were performed on IPEC-J2 cell monolayers grown in 12-well transwell plates (Corning Inc. Corning, NY, USA). IPEC-J2 cells (5 × 10^4^ cells/μL) were seeded into 1.12 cm^2^ Transwell-COL inserts with 0.4 μm pore membranes (Corning Inc.). The medium (400 µL in the insert and 1500 µL in the well) was changed daily. Electrical resistance was measured using the Millicell Electrical Resistance System (Millipore Corp, Billerica, MA, USA). On day eight post-differentiation, the TEER values reached steady-state. The TEER values of the IPEC-J2 cell monolayers were measured at 24 h after the addition of different concentrations of sodium succinate. Each well was measured in three different directions, and the TEER value was calculated in ohms using the formula (R_sample_ − R_blank_) × membrane area (cm^2^).

### 2.8. RNA Extraction and Real-Time PCR

The total RNA in the jejunal tissue and IPEC-J2 cells was extracted using the RNApure Total RNA kit (Aidlab, Beijing, China) according to the manufacturer’s instructions. The RNA concentrations and purity were measured by using a Nano-Drop spectrophotometer (ThermoFisher Scientific, Wilmington, DE, USA). The absorption ratio (OD260/OD280 nm) of the samples was between 1.8 and 2.0, which demonstrated a high purity of the RNA. The extracted RNA was reverse transcribed using a PrimeScriptTM RT Reagent Kit with gDNA Eraser (Takara Bio, Otsu, Japan) to generate cDNA. Quantitative real-time PCR (qPCR) for the relevant genes was performed with an ABI 7300 Real-Time qPCR system (Applied Biosystems, Foster, CA, USA) with fluorescence detection of SYBR green dye. The primers for qPCR are listed in Table 1. The relative expression of the genes was calculated using the formula 2^-ΔΔCt^.

### 2.9. Western Blot Analysis

Total proteins were extracted from the jejunum tissue samples and IPEC-J2 cells using the RIPA lysis buffer (Thermo Scientific, Waltham, MA, USA) containing protease inhibitors. The protein concentrations were determined using the BCA protein assay kit (Nanjing Jiancheng Institute of Bioengineering, Nanjing, China). All of the extracted proteins were diluted with the SDS loading buffer and boiled at 95 °C for 10 min. A total of 40 µg extracted protein were separated by 12% SDS-PAGE gel electrophoresis and then transferred onto the polyvinylidene fluoride (PVDF) membrane (Millipore, Bedford, MA, USA). The membranes were blocked with 5% milk for at least 1 h at room temperature, and then were incubated with the primary antibodies against ZO-1, Occludin, Claudin-1, and β-actin (Proteintech, Chicago, IL, USA) overnight at 4 °C. The membranes were washed four times with 1 × Tris-buffered saline-Tween (TBST) for 10 min and incubated with the anti-mouse (or rabbit) IgG HRP-conjugated secondary antibody (1:3000; Cell Signaling Technology, Danvers, USA) for 1 h. After washing with 1 × TBST three times, the target protein bands were visualized with the electrochemiluminescence visualized system (Tanon, Shanghai, China). Band intensities were quantified using the ImageJ version 1.51 software [20], and all results were expressed as the target protein/β-actin protein ratio.

### 2.10. ELISA Assays

Protein levels of GPR91 and inflammatory cytokines, IL-6, IL-8, IL-10, IL-18, and IL-25, were measured by Enzyme Linked Immunosorbent Assay (ELISA) using commercially available kits (Porcine GPR91, Porcine IL-6, Porcine IL-8, Porcine IL-10, Porcine IL-18, and Porcine IL-25 Quantikine ELISA kits; Nanjing Jiancheng Institute of Bioengineering, Nanjing, China) according to the manufacturer’s instructions.

### 2.11. Statistical Analyses

The statistical analyses were performed in SPSS 20.0 (SPSS Inc., Chicago, IL, USA) and the graphs were generated using Graphpad Prism (La Jolla, CA, USA). The data were analyzed by one-way analysis of variance (ANOVA) and Duncan’s multiple comparison to evaluate differences between the treatments. Different superscript letters in the same row (a,b) indicate significant differences, which were considered significant at *p* < 0.05. The results are expressed as means and standard errors of measurement (SEMs).

## 3. Results

### 3.1. Dietary Succinate Supplementation Improves Intestinal Morphology in Growing Pigs

**Figure 1:**
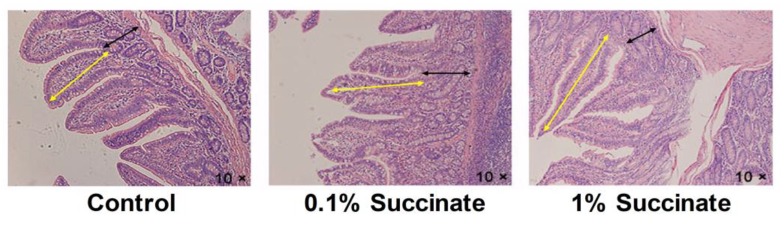
Histological evaluation of jejunal tissues from pig fed with basal, 0.1% succinate, and 1% succinate diet by hematoxylin and eosin (H&E)-staining. The yellow arrows denote villi length. The black arrows denote crypt depth.

We first evaluated the effects of different levels of sodium succinate on the intestinal development of growing pigs. The lengths and relative weights of the jejunum, ileum, and colon were measured and are shown in Table 2 and Table 3. No significant differences were observed in the lengths and relative weights of the jejunum, ileum, and colon between the three groups (*p* > 0.05). The intestinal morphological characteristics are shown in Figure 1 and Table 4. The diet supplemented with 1% sodium succinate increased the villi height and decreased the crypt depth in the jejuna of pigs (*p* < 0.05). The jejunal villi height/crypt depth ratio in the 1% succinate group was higher than that of the control group (*p* < 0.05). There was no obvious difference in the villi height, crypt depth, and villi height/crypt depth of the ilea between the three groups (*p* > 0.05).

### 3.2. Succinate Promotes Intestinal Epithelial Integrity

**Figure 2:**
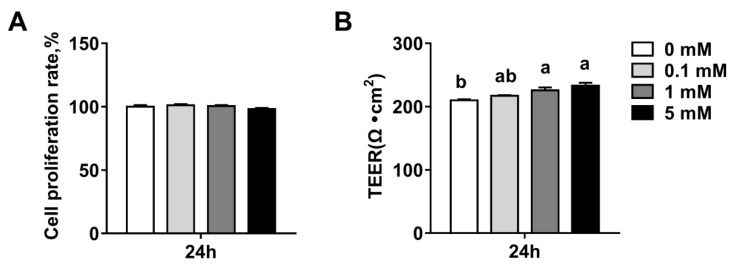
Effects of sodium succinate on intestinal permeability in intestinal porcine epithelial cell (IPEC)-J2 cells: (**A**) cell proliferation rate and (**B**) trans-epithelial electrical resistance (TEER). Different superscript letters indicate significant differences between groups (*p* < 0.05).

To determine whether succinate affected the integrity of intestinal epithelium, we examined the effect of succinate on epithelial permeability by measuring the TEER in IPEC-J2 cells. We first confirmed that a succinate concentration range from 0.1 mM to 5 mM did not cause a significant reduction in the viability of the IPEC-J2 cells (*p* > 0.05) (Figure 2A). As shown in Figure 2B, 1 mM and 5 mM succinate treatment significantly increased the TEER (*p* < 0.05) at 24 h (Figure 2B), indicating that succinate decreased intestinal epithelial permeability.

### 3.3. Succinate Promotes the Expression of Tight Junction Proteins in the Jejunum

**Figure 3:**
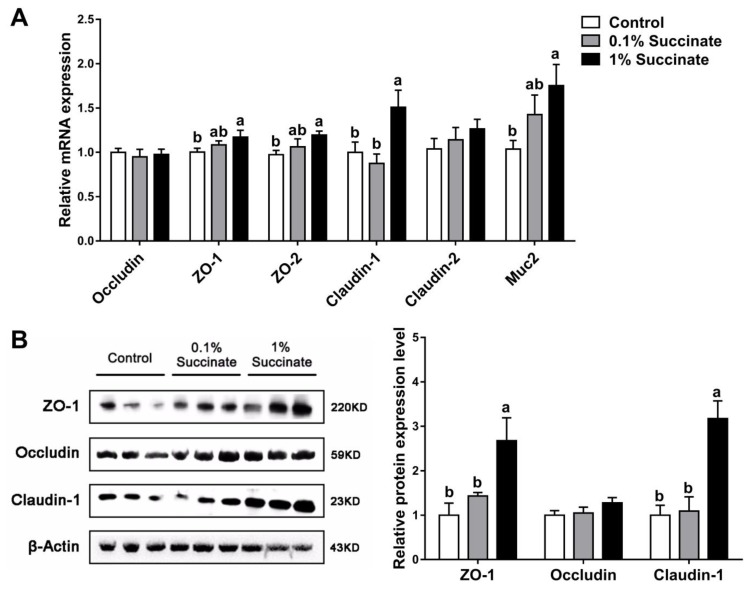
Effects of diet sodium succinate supplementation on the expression of barrier proteins in the jejunum of pigs: (**A**) relative mRNA levels and (**B**) the bands and relative band density of the proteins. Different superscript letters indicate significant differences between groups (*p* < 0.05).

We further assessed the effects of different levels of succinate on the expression of barrier-related proteins in the pig jejuna by qPCR and Western blotting analysis. Compared with the control group, pigs fed 1% sodium succinate had greater mRNA levels of the tight junction proteins ZO-1, ZO-2, and claudin-1, and the mucin protein Muc2 in jejunal tissue (*p* < 0.05) (Figure 3A). The protein levels of ZO-1 and claudin-1 were also increased (Figure 3B). No significant differences (*p* > 0.05) were observed in the expression of tight junction proteins between the 0.1% sodium succinate supplemented group and the control group.

**Figure 4:**
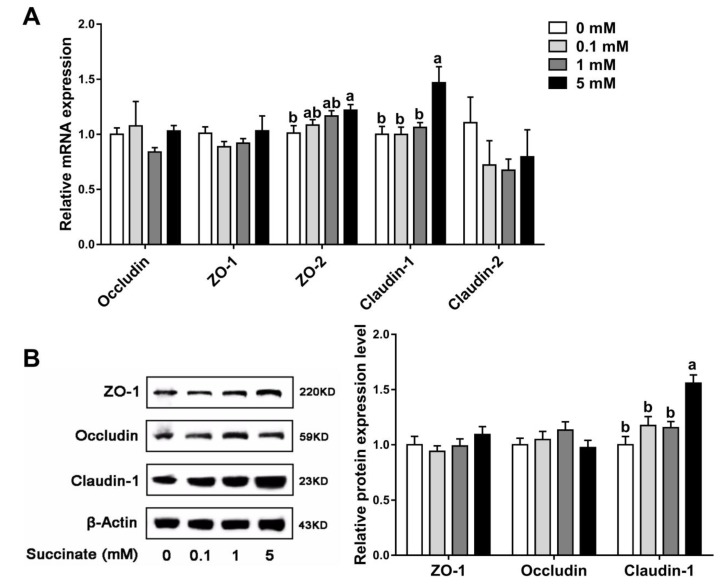
Effects of sodium succinate at different levels on the expression of tight junction proteins in IPEC-J2 cells: (**A**) relative mRNA levels and (**B**) the bands and relative band density of the proteins. Different superscript letters indicate significant differences between groups (*p* < 0.05).

Accordingly, in vitro, higher mRNA levels of claudin-1 and ZO-2 were observed in cells treated with 5 mM sodium succinate (*p* < 0.05) than in control cells, but no significant changes were detected between the other treatment groups and the control group (*p* > 0.05) (Figure 4A). Meanwhile, the increased level of claudin-1 protein in the 5 mM sodium succinate treatment group was confirmed by Western blotting (Figure 4B). These results demonstrated that succinate improved intestinal epithelial barrier function by upregulating several tight junction proteins.

### 3.4. Succinate Induces Inflammatory Cytokine Expression in the Jejunum

To evaluate the effects of succinate on intestinal inflammation, we quantitated the expression of inflammatory cytokines by qPCR analysis in the jejuna of growing pigs. The results showed that dietary supplementation with 1% sodium succinate increased the mRNA levels of IL-25, IL-10, IL-8, and IL-18 in the jejunum compared with the control group (*p* < 0.05), whereas no significant changes were observed (*p* > 0.05) between the 0.1% succinate-supplemented group and the control group (Table 5). Moreover, the ELISA results confirmed that 1% sodium succinate treatment significantly increased the protein concentrations of IL-25, IL-8, IL-10, and IL-18 (*p* < 0. 05), but had no significant effect on the concentration of IL-6 (*p* > 0.05) (Table 6).

We next assessed the effects of different concentrations of succinate on the expression of inflammatory cytokines in IPEC-J2 cells. In contrast to the in vivo experiment, succinate treatment did not affect the expression of IL-10 in vitro (undetectable of IL-25), whereas 5 mM sodium succinate significantly upregulated IL-6, IL-8, and IL-18 mRNA expression (*p* < 0.05) (Table 7). The ELISA results also showed higher protein levels of IL-6, IL-8, and IL-18 in the succinate-treated groups compared with the control group (Table 8). Collectively, these results demonstrated that succinate positively regulated the expression of several inflammatory cytokines in the jejunum.

### 3.5. Succinate Activates the Succinate Receptor GPR91 in the Jejunum

**Figure 5:**
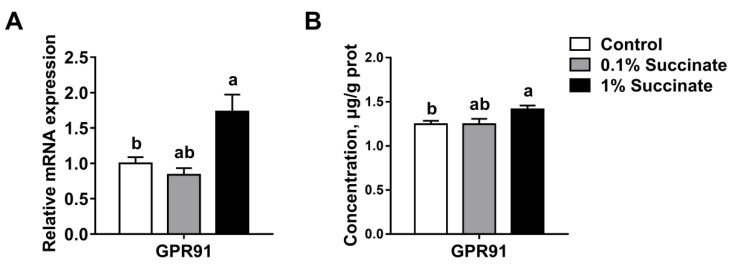
Effects of sodium succinate on the mRNA expression (**A**) and protein concentration (**B**) of GPR91 in the jejunum of pigs.

Recent studies have shown that succinate regulated intestinal inflammation and immunity through GPR91 signaling [16,17]. Thus, we measured GPR91 expression in the jejuna from succinate-supplemented and control pigs. The results showed that 1% succinate treatment significantly increased the mRNA level of GPR91 compared with treatment with 0.1% succinate and the controls (*p* < 0.05) (Figure 5A). Moreover, GPR91 protein expression was also higher in pigs supplemented with 1% succinate than in the other two groups, which was confirmed by the ELISA assays (*p* < 0.05) (Figure 5B). Taken together, these results indicate that succinate activated the expression of its receptor, GPR91, in pigs.

## 4. Discussion

In this study, we evaluated the effects of succinate on the intestinal epithelial barrier and inflammatory responses in pigs. The results showed that the intestinal morphology of pigs fed a diet supplemented with 1% succinate was altered. Furthermore, 5 mM succinate treatment increased the TEER in IPEC-J2 cells. In vivo and in vitro experiments revealed that succinate treatment upregulated the expression of tight-junction proteins claudin-1, ZO-1, and ZO-2 and modulated the secretion of several inflammatory cytokines in the jejunum. These findings identify a novel role of succinate in the modulation of intestinal epithelial barrier function.

The small intestinal epithelium consists of numerous finger-like luminal protrusions called villi and flask-shaped submucosal invaginations known as crypts [21]. Severe structural damage of the small intestine villi and crypts influences the epithelial barrier and absorption function of the small intestine [22]. While succinate has been shown to inhibit colonic cell proliferation and reduce the crypt depth in rats [23], the results of the current study showed that supplementation with 1% succinate significantly improved the villi height and the ratio of villi height and crypt depth in pigs. It also decreased the jejunal crypt depth. Similarly, several other metabolites, such as SCFAs, have been shown to improve small intestine morphology and function [9,24,25]. Growing evidence suggests that the integrity of the intestinal epithelial barrier is required for gut health [26]. Transepithelial electrical resistance is an important indicator of the integrity of the intestinal epithelial barrier. Previous studies have reported that SCFAs, particularly butyrate, promoted epithelial integrity by increasing the TEER in IPEC-J2 and Caco-2 cells [11,27]. In the present study, we found that treatment with 5 mM sodium succinate reduced cell permeability by increasing the TEER in IPEC-J2 cells. These results suggest that succinate protected the integrity of the small intestinal epithelium.

Tight junctions between epithelial cells are the most important components of the intestinal epithelial barrier. Tight junction complexes, composed of claudins, zona occludens, and occludin, maintain intestinal barrier integrity and regulate intestinal permeability. In the current study, supplementation with 1% succinate upregulated the expression of the claudin-1, ZO-1, and ZO-2 tight junction proteins in pigs. Consistent with the in vivo trial, treatment with 5 mM succinate increased the expression of claudin-1 and ZO-2 in IPEC-J2 cells. As the main structural and functional component of tight junctions, claudin-1 selectively prevents the passage of luminal substances through the intestinal epithelial barrier [28]. ZO-1 anchors claudins and occludin to intracellular actin, mediating crosslinks between the transmembrane proteins and the actin cytoskeletons [29]. ZO-1 and its closely related family member, ZO-2, regulate tight junction assembly and play crucial roles in maintaining epithelial permeability [30]. In Caco-2 cells, severe inflammation reduces the expression of ZO-1 and impairs epithelial barrier function [31]. Monteiro et al. found that ZO-2 directly interacts with junctional adhesion molecule-A (JAM-A) and is necessary for regulating epithelial barrier function [32]. Therefore, the increased TEER induced by succinate in vivo and in vitro may be attributed to the upregulation of claudin-1, ZO-1, and ZO-2. Moreover, the present study found that the mRNA expression of jejunal Muc2 was increased in pigs supplemented with 1% succinate compared with the control. Muc2 is largely produced by goblet cells in the small intestine, and serves as an important structural component of intestinal mucin layer to protect against the infection of luminal viruses [33,34]. Therefore, the upregulated Muc2 expression might improve the defense function of intestinal epithelial barrier. In general, succinate promotes intestinal epithelial barrier via selectively regulating the expression of tight junction proteins and mucin protein Muc2.

Intestinal epithelial integrity is related to immune response. Previous studies have revealed that succinate not only potentiated type 1 immunity through activating dendritic cells and macrophages, but also initiated type 2 immune responses by stimulating intestinal tuft cells [18,35,36]. In the present study, we assessed the effects of succinate on intestinal inflammatory cytokines in vivo and in vitro. Pigs fed 1% succinate had greater mRNA expression and protein concentrations of IL-25 and IL-10 in the jejunum compared with the control. IL-25 can be largely produced by intestinal mucosal CD4^+^ T cells in mouse [5]. Moreover, IL-25 in the small intestine is also made by tuft cells and activates type 2 innate lymphoid cells (ILC2s) to secrete IL-13, which triggers a type 2 immunity response, including goblet and tuft cell hyperplasia [37,38]. Consistent with our findings, a recent study showed that dietary succinate supplementation significantly stimulated the expression of IL-25 and induced IL-25 mediated type 2 immunity in the jejuna in mice [17]. IL-10 is an anti-inflammatory cytokine promoting tissue repair in type 2 responses [39], which is produced in large amount by intestinal immune cells, including dendritic cells, macrophages, natural killer (NK) cells, neutrophils, and CD4^+^ T cells [40,41]. Differently, in the current study, succinate supplementation did not affect IL-Iβ expression in the jejunum of pigs, although succinate has been shown to enhance IL-1β production during inflammation in bone-marrow-derived macrophages [42]. We also found that 1% succinate can evoke an inflammatory response via the upregulation of IL-8 mRNA expression and protein secretion. IL-8 secreted by intestinal epithelial cells has an important role in neutrophil recruitment. Similarly, butyrate has also been shown to induce IL-8 secretion in IPEC-J2 and Caco-2 cells [11,43]. Several pro-inflammatory and anti-inflammatory cytokines were both upregulated by succinate, which suggests that succinate activated innate immunity and enhanced the immune function of the intestine.

In vitro, succinate treatment did not affect the expression of IL-10, but increased the expression of IL-6, IL-8, and IL-18. IL-6 is an inflammatory cytokine involved in the development of Th17 cells [44]. Kuhn et al. showed that IL-6 derived from intraepithelial lymphocytes enhanced intestinal epithelial cell proliferation and contributed to mucosal injury healing [45]. IL-18 was increased in Specific Pathogen Free (SPF) mice treated with 200 mM and 300 mM acetate, and contributes to the maintenance of intestinal epithelial integrity, repair, and gut homeostasis [46,47]. We also previously showed that propionate upregulates the expression of IL-18 in the gut [12]. Therefore, the increased expression of inflammatory cytokines IL-6 and IL-18 suggests that tight-junction integrity was promoted by succinate.

Numerous studies have demonstrated that succinate regulated intestinal immunity through its cognate receptor GPR91 [16,17], which is a G protein-coupled receptor widely-expressed in macrophages, dendritic cells, and the small intestine [48]. In the current study, we found that diet supplemented with 1% succinate significantly increased the mRNA level of GPR91 in the jejuna of pigs. Further, the ELISA assay results also demonstrated that the protein concentration of GPR91 was increased by succinate in the jejuna of pigs. We speculate that succinate improved epithelial barrier integrity via GPR91 signaling, but the exact mechanism of this effect requires further study.

## 5. Conclusions

In conclusion, succinate improved intestinal morphology and decreased the intestinal epithelial permeability, as shown by the increase in tight-junction proteins claudin-1, ZO-1, and ZO-2 in vivo and in vitro. Furthermore, succinate affected intestinal immune responses by regulating several inflammatory cytokines. This study identifies a novel role for succinate in the modulation of intestinal epithelial barrier function, which may be a nutritional target to improve gut health in animals.

## Figures and Tables

**Table 1:** List of PCR primers.

Gene	Forward(5′-3′)	Reverse(5′-3′)
*β-actin*	F: CCACGAAACTACCTTCAACTC	R: TGATCTCCTTCTGCATCCTGT
*Claudin-1*	F: AGATTTACTCCTACGCTGGT	R: GCACCTCATCATCTTCCAT
*Claudin-2*	F: CTCGTTGGCCTGTATCATCACC	R: CAGGGGGGAGTAGAAGTCCC
*Occludin*	F: ATGCTTTCTCAGCCAGCGTA	R: AAGGTTCCATAGCCTCTCGGTC
*ZO-1*	F: GAGGATGGTCACCGTGGT	R: GGAGGATGCTGTTGTCTCGG
*ZO-2*	F: GCAGAGACAACCCCCACTTT	R: CGTTAACCATGACCACCCGA
*Muc2*	F: CTGCTCCGGGTCCTGTGGGA	R: CCCGCTGGCTGGTGCGATAC
*TNF-α*	F: CCACGCTCTTCTGCCTACTGC	R: GCTGTCCCTCGGCTTTGAC
*IL-1β*	F: AGTGGAGAAGCCGATGAAGA	R: CATTGCACGTTTCAAGGATG
*IL-4*	F: GCTGCCCCAGAGAACACGAC	R: AGGTTCCTGTCAAGTCCGCTC
*IL-6*	F: CCTCTCCGGACAAAACTGAA	R: TCTGCCAGTACCTCCTTGCT
*IL-8*	F: TAGGACCAGAGCCAGGAAGA	R: AGCAGGAAAACTGCCAAGAA
*IL-10*	F: AGGTTCCTGTCAAGTCCGCTC	R: GCCAGGAAGATCAGGCAATA
*IL-13*	F: AAGTGGCCCAGTTCGTAAAAGA	R: ACCCGTGGCGAAAAATCA
*IL-18*	F: TATGCCTGATTCTGACTGTT	R: ATGAAGACTCAAACTGTATCT
*IL-25*	F: GCCCCTTGGAGATACGAGTT	R: CGGTAGAAGACGGTCTGGTT
*GPR91*	F: GATTGAGTTCATTGTGGGA	R: CTGGTGTAGAGGTTGGCAT

IL: interleukin, GRP: G protein-coupled receptor, ZO: zona occludens protein, Muc: mucin, TNF: Tumor Necrosis Factor.

**Table 2:** Effects of diet sodium succinate supplementation on the length of different intestinal segments in growing pigs.

Item	Control	0.1% Succinate	1% Succinate	*p*-Value
Jejunum (cm)	1447.50 ± 79.46	1505.00 ± 82.76	1313.75 ± 66.65	0.218
Ileum (cm)	37.63 ± 2.15	35.88 ± 2.30	35.13 ± 1.95	0.702
Colon (cm)	278.13 ± 17.55	278.75 ± 16.19	257.50 ± 11.61	0.546

**Table 3:** Effects of diet sodium succinate supplementation on the intestinal index in growing pigs.

Item	Control	0.1% Succinate	1% Succinate	*p*-Value
Relative weight of jejunum (%)	4.90 ± 0.35	4.63 ± 0.11	4.44 ± 0.07	0.343
Relative weight of ileum (%)	0.17 ± 0.02	0.19 ± 0.02	0.19 ± 0.01	0.499
Relative weight of colon (%)	3.10 ± 0.28	3.39 ± 0.17	3.28 ± 0.32	0.733

**Table 4:** Effect of diet sodium succinate supplementation on the intestinal morphology in growing pigs.

Item	Control	0.1% Succinate	1% Succinate	*p*-Value
**Jejunum**				
Villus height (µm)	378.52 ± 24.18 ^b^	395.41 ± 22.14 ^b^	470.57 ± 26.05 ^a^	0.038
Crypt depth (µm)	326.98 ± 27.68 ^a^	283.81 ± 21.54 ^a^	236.44 ± 19.17 ^b^	0.045
Villus height/Crypt depth	1.37 ± 0.24 ^b^	1.44 ± 0.12 ^b^	2.18 ± 0.16 ^a^	0.010
**Ileum**				
Villus height (µm)	314.85 ± 23.61	290.41 ± 15.87	340.14 ± 16.59	0.205
Crypt depth (µm)	242.69 ± 15.00	317.59 ± 40.36	309.57 ± 33.74	0.208
Villus height/Crypt depth	1.40 ± 0.19	1.03 ± 0.11	1.16 ± 0.09	0.183

Different superscript letters in the same row indicate significant differences between groups (*p* < 0.05).

**Table 5:** Effects of diet sodium succinate supplementation on the mRNA levels of inflammatory cytokines in the jejunum of pigs.

Gene	Control	0.1% Succinate	1% Succinate	*p*-Value
IL-25	1.00 ± 0.20	1.46 ± 0.19 ^ab^	2.14 ± 0.39 ^a^	0.027
IL-13	1.00 ± 0.19	0.91 ± 0.10	0.96 ± 0.08	0.899
IL-1β	1.00 ± 0.09	0.85 ± 0.10	0.85 ± 0.24	0.740
TNF-α	1.00 ± 0.14	1.11 ± 0.14	1.36 ± 0.32	0.462
IL-4	1.00 ± 0.25	0.87 ± 0.06	0.83 ± 0.15	0.754
IL-6	1.00 ± 0.10	1.01 ± 0.19	1.11 ± 0.23	0.906
IL-10	1.00 ± 0.08 ^b^	1.12 ± 0.11 ^b^	1.70 ± 0.38 ^a^	0.043
IL-8	1.00 ± 0.10 ^b^	1.01 ± 0.10 ^b^	1.36 ± 0.12 ^a^	0.041
IL-18	1.00 ± 0.15 ^b^	1.07 ± 0.09 ^ab^	1.39 ± 0.11 ^a^	0.077

Different superscript letters in the same row indicate significant differences between groups (*p* < 0.05).

**Table 6:** Effects of diet sodium succinate supplementation on the protein abundances of inflammatory cytokines in the jejunum of pigs.

Item	Control	0.1% Succinate	1% Succinate	*p*-Value
IL-25, ng/g prot	24.43 ± 1.06 ^b^	24.87 ± 1.27 ^b^	28.79 ± 1.36 ^a^	0.041
IL-10, ng/g prot	6.61 ± 0.35 ^b^	6.96 ± 0.36 ^ab^	7.78 ± 0.20 ^a^	0.073
IL-6, ng/g prot	31.68 ± 1.23	34.22 ± 1.95	31.72 ± 2.13	0.569
IL-8, ng/g prot	2.97 ± 0.17 ^b^	3.04 ± 0.12 ^ab^	3.39 ± 0.09 ^a^	0.081
IL-18, ng/g prot	4.97 ± 0.27 ^b^	5.82 ± 0.19 ^ab^	6.20 ± 0.37 ^a^	0.021

Different superscript letters in the same row indicate significant differences between groups (*p* < 0.05).

**Table 7:** Effects of sodium succinate at different levels on the mRNA levels of inflammatory cytokines in IPEC-J2 cells.

Gene	Succinate Treatment				*p-*Value
	0 mM	0.1 mM	1 mM	5 mM	
*IL-6*	1.00 ± 0.04 ^b^	1.09 ± 0.06 ^ab^	1.07 ± 0.04 ^ab^	1.23 ± 0.06 ^a^	0.027
*IL-8*	1.00 ± 0.09 ^b^	0.97 ± 0.06 ^b^	1.14 ± 0.05 ^ab^	1.26 ± 0.08 ^a^	0.038
*IL-10*	1.00 ± 0.28	0.99 ± 0.27	0.65 ± 0.21	0.85 ± 0.20	0.700
*IL-18*	1.00 ± 0.09 ^b^	1.00 ± 0.09 ^b^	1.23 ± 0.11 ^ab^	1.40 ± 0.10 ^a^	0.032
*TNF-α*	1.00 ± 0.08	1.06 ± 0.18	1.00 ± 0.07	1.05 ± 0.04	0.970

Different superscript letters in the same row indicate significant differences between groups (*p* < 0.05).

**Table 8:** Effects of sodium succinate at different levels on the protein abundances of inflammatory cytokines in IPEC-J2 cells.

Item	Succinate Treatment				*p*-Value
	0 mM	0.1 mM	1 mM	5 mM	
IL-6, ng/L	171.93 ± 3.38 ^b^	191.33 ± 3.25 ^a^	172.15 ± 4.91 ^b^	188.33 ± 2.87 ^a^	0.003
IL-8, ng/L	16.95 ± 0.52 ^b^	18.66 ± 0.56 ^a^	19.82 ± 0.67 ^a^	19.95 ± 0.29 ^a^	0.002
IL-18, ng/L	30.79 ± 1.08 ^b^	32.67 ± 1.27 ^ab^	32.97 ± 1.24 ^ab^	34.88 ± 0.49 ^a^	0.092

Different superscript letters in the same row indicate significant differences between groups (*p* < 0.05).
